# Safety Analysis of Apatinib Combined with Chemotherapy in the Treatment of Advanced Gastric Carcinoma: A Randomised Controlled Trial

**DOI:** 10.1155/2021/5177140

**Published:** 2021-08-09

**Authors:** Min Yuan, Zhaohui Wang, Yibo Zhang, Liying Chen, Yuting Liu, Cui Cui, Bo Sun

**Affiliations:** ^1^Department of Nursing, Weifang People's Hospital, Weifang 261041, China; ^2^Department of Liver Disease, Qingdao No. 6 People's Hospital, Qingdao 266033, China; ^3^Department of Wound and Ostomy Outpatient, Weifang People's Hospital, Weifang 261041, China; ^4^Department of Dentistry, Weifang People's Hospital, Weifang 261041, China; ^5^Ward 2, Department of Neurology, Yidu Central Hospital of Weifang, Qingzhou 262500, China; ^6^Department of Vascular Surgery, Weifang People's Hospital, Weifang 261041, China

## Abstract

**Objective:**

To study the safety of apatinib combined with chemotherapy in the treatment of advanced gastric carcinoma (GCA).

**Methods:**

74 patients with advanced GCA treated in the oncology department of Weifang People's Hospital (January 2019–January 2020) were enrolled in this study and equally split into study group (SG) and reference group (RG) according to the odd and even admission numbers. RG underwent chemotherapy alone, while SG received apatinib combined with chemotherapy. The clinical indicators of serum matrix metalloproteinase 9 (MMP-9), serum interleukin-2 receptor (SIL-2R), and immune cell level were detected in the two groups before and after treatment to analyze the therapeutic effect of different treatment methods on patients with advanced gastric carcinoma.

**Results:**

No obvious differences in gender ratio, average age, average BMI, pathological staging, pathological types, organ metastasis types, and residence were observed between the two groups (*P* > 0.05). The short-term follow-up results showed that the disease control rate (DCR) in SG was markedly higher compared with RG (*P* < 0.05). The MMP-9 and SIL-2R levels in both groups after treatment decreased (*P* < 0.05), and the levels in SG after treatment were notably lower compared with RG (*P* < 0.001). Compared with RG, CD3^+^, CD4^+^, and CD4^+^/CD8^+^ levels in SG after treatment were notably higher (*P* < 0.001), while the CD8^+^ level was notably lower (*P* < 0.001). The median progression-free survival (MPFS) and overall survival (OS) in SG were markedly higher compared with RG (*P* < 0.001). The GQOLI-74 scores in both groups after treatment increased (*P* < 0.001), and the GQOLI-74 score in SG after treatment was markedly higher compared with RG (*P* < 0.001). The total incidence of adverse reactions was lower in SG than in RG (*P* < 0.05).

**Conclusion:**

Apatinib combined with chemotherapy is superior to chemotherapy alone in effectively improving treatment outcomes in patients with advanced GCA.

## 1. Introduction

Gastric carcinoma (GCA) is a common tumor disease in gastroenterology. China is a country with a high incidence of GCA, and about 350,000 people die from GCA every year. Therefore, GCA has become the main cancer that endangers human life and health [1–3]. In recent years, due to unreasonable diet structure, high working pressure, chronic atrophic gastritis, and other reasons, GCA patients tend to be younger. Since the early clinical symptoms of GCA are not obvious, most patients have missed the best treatment opportunity. Chemotherapy is the main treatment for prolonging the survival period of patients at present, and there is no standard scheme for chemotherapy. Oxaliplatin is widely applied to slow down the disease progression and relieve the clinical symptoms, with good short-term curative effect and prognosis [4–7]. However, chemotherapy can not only cause strong side effects and increase the pain of treatment but also increase the drug resistance of tumor cells, resulting in poor efficacy. Studies have found that apatinib is a highly selective tyrosinase inhibitor acting on vascular endothelial growth factor 2 (VEGFR-2), and its efficacy and safety in advanced pancreatic cancer have been confirmed [8–10]. Based on this, the paper aims to investigate the safety of apatinib combined with chemotherapy for treating patients with advanced GCA.

## 2. Materials and Methods

### 2.1. General Information

74 patients with advanced GCA treated in the oncology department of Weifang People's Hospital (January 2019–January 2020) were enrolled in this study, and equally split into the study group (SG) and reference group (RG) according to the odd and even admission numbers.

### 2.2. Inclusion Criteria

1. All enrolled patients met the diagnostic criteria of advanced GCA in Guidelines for Diagnosis and Treatment of Primary Gastric Cancer [10] of Chinese Society of Clinical Oncology (CSCO) and confirmed by clinical diagnosis and imaging.
2. The expected survival period was more than 3 months.
3. The patients had normal blood routine, electrolyte, and liver function with no contraindication to chemotherapy.
4. Eastern Cooperative Oncology Group (ECOG) score was 0–2.
5. This study got the approval of the ethics committee of Weifang people's Hospital, and the patients had signed the informed consent.

### 2.3. Exclusion Criteria

1. The patients had other malignant tumors
2. The blood pressure of the patients could not be controlled
3. The patients were receiving anticoagulant therapy or thrombolytic therapy
4. The patients had a contraindication to apatinib
5. The patients did not recover from the adverse reactions of early chemotherapy

### 2.4. Methods

RG was treated with chemotherapy alone, with the regimen of 5-fluorouracil (5-Fu), oxaliplatin, and calcium folinate (FOLFOX6 regimen or its improved regimen). The patients received 400 mg/m^2^ of 5-Fu (manufacturer: Shanghai Acmec Biochemical Co., Ltd; art. no : F93580-100 g) by intravenous drip on the 1st day, with 2400–3000 mg/m^2^ of continuous intravenous drip for 46 hours. The patients received 80–95 mg/m^2^ of oxaliplatin (SFDA approval no.: H20143023; manufacturer: Hainan Jinrui Pharmaceutical Co., Ltd; specification: 50 mg) by intravenous drip for 3 hours on the 1st day. The patients received 200 mg/m^2^ of calcium folinate (SFDA approval no.: H20040612; manufacturer: Guangdong Lingnan Pharmaceutical Co., Ltd.; specification: 0.1 g) by intravenous drip on the 1st day, with 14 days as a cycle. On this basis, SG was given oral apatinib (SFDA approval no.: H20140103; manufacturer: Jiangsu Hengrui Pharmaceutical Co., Ltd.; specification: 0.25 g × 10 tablets/box) at a dose of 250–850 mg/d. 14 days was a course of treatment, and 6 courses were performed.

### 2.5. Observation Indexes

#### 2.5.1. Disease Control Rate

The response evaluation criteria in solid tumor (RECIST1.1) [11] was applied to evaluate the efficacy in the two groups. (1) Complete response (CR): the tumor disappeared completely. (2) Partial response (PR): the maximum tumor diameter decreased by 30%. (3) Stable disease (SD): the change of diameter was between PR and PD. (4) Progressive disease (PD): the diameter increased by 20% or new lesions were found. Disease control rate (DCR) = CR + PR + SD.

3 ml of fasting venous blood was collected from patients before and after treatment. The upper serum was taken after centrifugation, and the MMP-9 and SIL-2R levels were determined by ELISA. The kits were purchased from Jiangsu Jingmei Biotechnology Co., Ltd.

#### 2.5.2. Immune Function

The FACSVia flow cytometer (manufacturer: Shanghai Huanxi Medical Equipment Co., Ltd.) was used to measure the CD4^+^, CD8^+^, CD3^+^, and CD4^+^/CD8^+^ levels before and after treatment.

The patients were followed up until July 2020, and returned to the hospital for magnetic resonance imaging and computed tomography after one month. The median progression-free survival (MPFS) and overall survival (OS) of patients were recorded.

Generic Quality of Life Inventory-74 (GQOLI-74) [12] was used to score the quality of life (QOL) before and after intervention, including psychological function, somatic function, social function, and material life. Higher scores represented better QOL.

The incidence of adverse reactions during treatment was recorded, including alimentary tract hemorrhage, myelosuppression, liver function damage, skin and mucosal reaction, and thrombocytopenia.

### 2.6. Statistical Methods

All the experimental data were processed by SPSS21.0 software and graphed by GraphPad Prism 7 (GraphPad Software, San Diego, USA). The count data were tested by *X*^2^, expressed by (*n* (%)), and the measurement data were measured by *t*-test, expressed by (‾*x* ± *s*). The differences were statistically significant at *P* < 0.05.

## 3. Results

### 3.1. Comparison of Clinical Data

No obvious differences in gender ratio, average age, average BMI, pathological staging, pathological types, organ metastasis types, and residence were observed between the two groups (*P* > 0.05), as shown in Table 1.

### 3.2. Comparison of Short-Term Efficacy

The short-term follow-up results showed that the DCR in SG was markedly higher compared with RG (*P* < 0.05), as shown in Table 2.

### 3.3. Comparison of Serum Indexes before and after Treatment

**Figure 1:**
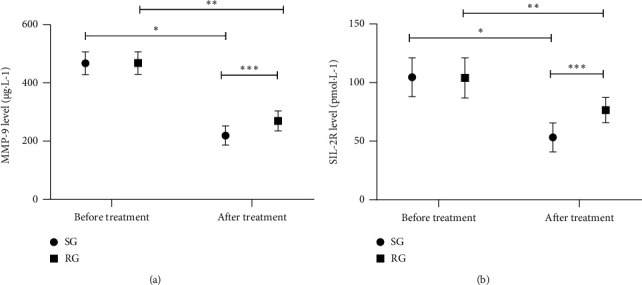
Comparison of serum indexes between the two groups before and after treatment (‾*x* ± *s*). (a) Comparison of MMP-9 levels before and after treatment. The abscissa represents before treatment and after treatment, and the ordinate represents the MMP-9 level (*μ*g·L^−1^). The MMP-9 levels in SG before and after treatment were (467.34 ± 39.56) *μ*g·L^−1^ and (219.46 ± 32.54) *μ*g·L^−1^, respectively. The MMP-9 levels in RG before and after treatment were (468.12 ± 38.74) *μ*g·L^−1^ and (269.56 ± 34.17) *μ*g·L^−1^, respectively. ^*∗*^indicated an obvious difference in the MMP-9 levels of SG before and after treatment (*t* = 29.436, *P* < 0.05). ^*∗∗*^indicated an obvious difference in the MMP-9 levels of RG before and after treatment (*t* = 23.381, *P* < 0.05). ^*∗∗∗*^indicated an obvious difference in the MMP-9 levels between the two groups after treatment (*t* = 6.459, *P* < 0.05). (b) Comparison of SIL-2R levels before and after treatment. The abscissa represented before treatment and after treatment, and the ordinate represented the SIL-2R level (pmol·L^−1^). The SIL-2R levels in SG before and after treatment were (104.57 ± 16.54) pmol·L^−1^ and (53.17 ± 12.34) pmol·L^−1^, respectively. The SIL-2R levels in RG before and after treatment were (103.92 ± 17.12) pmol·L^−1^ and (76.46 ± 10.83) pmol·L^−1^, respectively. ^*∗*^ indicated an obvious difference in the SIL-2R levels of SG before and after treatment (*t* = 15.151, *P* < 0.05); ^*∗∗*^indicated an obvious difference in the SIL-2R levels of RG before and after treatment (*t* = 8.245, *P* < 0.05); ^*∗∗∗*^indicated an obvious difference in the SIL-2R levels between the two groups after treatment (*t* = 8.629, *P* < 0.05).

The MMP-9 and SIL-2R levels in both groups after treatment decreased (*P* < 0.05), and the levels in SG after treatment were markedly lower compared with RG (*P* < 0.05), as shown in Figure 1.

### 3.4. Comparison of Immune Function after Treatment

Compared with RG, CD3^+^, CD4^+^, and CD4^+^/CD8^+^ levels in SG after treatment were notably higher (*P* < 0.001), while the CD8^+^ level was notably lower (*P* < 0.001), as shown in Table 3.

### 3.5. Comparison of Survival Period

The MPFS and OS in SG were markedly higher compared with RG (*P* < 0.001), as shown in Table 4.

### 3.6. Comparison of GQOLI-74 Scores before and after Treatment

**Figure 2:**
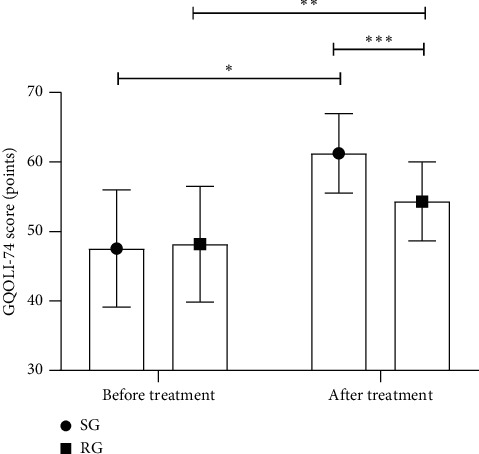
Comparison of GQOLI-74 scores before and after treatment (‾*x* ± *s*). *Note*. The abscissa represented before treatment and after treatment, and the ordinate represented the GQOLI-74 score (points). The GQOLI-74 scores in SG before and after treatment were (47.56 ± 8.45) and (61.24 ± 5.72), respectively. The GQOLI-74 scores in RG before and after treatment were (48.17 ± 8.32) and (54.33 ± 5.68), respectively. ^*∗*^indicated an obvious difference in the GQOLI-74 scores of SG before and after treatment (*t* = 8.155, *P* < 0.001); ^*∗∗*^indicated an obvious difference in the GQOLI-74 scores of RG before and after treatment (*t* = 3.719, *P* < 0.001); ^*∗∗∗*^indicated an obvious difference in the GQOLI-74 scores between the two groups after treatment (*t* = 5.214, *P* < 0.001).

The GQOLI-74 scores in both groups after treatment increased (*P* < 0.001), and the GQOLI-74 score in SG after treatment was notably higher compared with RG (*P* < 0.001), as shown in Figure 2.

### 3.7. Comparison of the Incidence of Adverse Reactions

The total incidence of adverse reactions in SG was markedly lower compared with RG (*P* < 0.05), as shown in Table 5.

## 4. Discussion

The pathological mechanism of GCA may be related to the following factors. (1) *Helicobacter pylori* infection: *Helicobacter pylori* infection leads to injury and apoptosis of gastrointestinal mucosal epithelial cells, increases oxygen free radicals, and cell proliferation and deterioration, eventually resulting in gastric cancer. (2) Life style and dietary habits: long-term eating of charcoal roasted or salted food increases the incidence of GCA. In addition, smoking is also the main factor leading to GCA. Chemotherapy is the main way to prolong the survival time of patients with advanced GCA, but there is no standard scheme for chemotherapy at present. The common chemotherapy drugs include antimicrotubule, fluorouracil, and platinum drugs, which can reduce the gastrointestinal reaction of patients to a certain extent and delay the disease progression [13, 14]. Clinical studies have found that abnormal angiogenesis is one of the basic features of malignant tumors and also one of the main ways of tumor progression [15]. Vascular endothelial growth factor (VEGF) plays an important role in the process of abnormal angiogenesis, which is mainly secreted by tumor cells or tumor stromal cells. During tumor enlargement, abnormal tumor vascular system causes the increased VEGF expression level, thus promoting the formation of abnormal angiogenesis. Therefore, the targeted therapy of VEGF has become a new method for the treatment of advanced GCA [16–18]. VEGFR-2 facilitates the proliferation of vascular endothelial cells by activating the MAPK signaling pathway. Apatinib, as an oral small molecule TKI against angiogenesis, can block VEGFR-2 in advanced GCA patients and reduce the activation of mitogen-activated protein kinase, thus inhibiting the proliferation of vascular endothelial cells [19–21].

As an interleukin receptor, SIL-2R can combine with IL-2 to reduce the activity of IL-2. If SIL-2R is highly expressed in serum, the cellular immunity induced by IL-2 will be inhibited, resulting in the decline of immune function and thereby accelerating the infiltration and proliferation of tumor cells [22–24]. This study confirmed that the serum SIL-2R level after apatinib combined with chemotherapy was markedly lower than that after chemotherapy alone, indicating that apatinib combined with chemotherapy reduces the serum SIL-2R level in advanced GCA patients, slows down the proliferation of tumor cells, and improves the prognosis. This study also found that the DCR in SG was markedly higher compared with RG, demonstrating that efficacy of the combined therapy in treating advanced GCA is markedly better than that of chemotherapy alone. Kano et al. [25] pointed out in their study that the disease control rate was 43.52% in the patients with lung cancer treated with chemotherapy alone, while that was 68.25% in the patients treated with apatinib combined with chemotherapy, suggesting that the effect of the combined therapy is better in treating lung cancer. In this study, both groups of patients had different types of adverse reactions during treatment, mainly including thrombocytopenia and alimentary tract hemorrhage. Most adverse reactions of patients could be relieved after symptomatic treatment, and some patients stopped medication due to intolerance. However, the incidence of adverse reactions of apatinib combined with chemotherapy was notably lower than that of chemotherapy alone, suggesting that apatinib combined with chemotherapy was safer than chemotherapy alone. This study also has some deficiencies such as the small size of selected samples. Therefore, the sample size should be further expanded for in-depth studies.

In conclusion, for advanced GCA patients, apatinib combined with chemotherapy has a high DCR with convenient and simple administration and can alleviate the clinical symptoms of patients with obvious clinical efficacy. Therefore, it is worth applying and promoting in clinic.

## Figures and Tables

**Table 1:** Comparison of clinical data.

Items	SG (*n* = 37)	RG (*n* = 37)	*χ*^2^/*t*	*P*
*Gender*			0.492	0.483
Male	22 (59.46%)	19 (51.35%)
Female	15 (40.54%)	18 (48.65%)
Average age (years old)	53.21 ± 6.51	53.25 ± 6.38	0.027	0.979
Average BMI (kg/m^2^)	22.84 ± 1.73	22.86 ± 1.78	0.049	0.961
*Pathological staging*			0.237	0.626
III	23 (62.16%)	25 (67.57%)
IV	14 (37.84%)	12 (32.43%)
*Pathological types*				
Poorly differentiated adenocarcinoma	16 (43.24%)	14 (37.84%)	1.458	0.227
Medium differentiated adenocarcinoma	17 (45.95%)	18 (48.65%)	0.046	0.831
Carcinoma mucocellulare	4 (10.81%)	5 (13.51%)	0.127	0.722
*Organ metastasis types*			0.237	0.626
Single organ metastasis	14 (37.84%)	12 (32.43%)
Multiple organ metastasis	23 (62.16%)	25 (67.57%)
*Residence*			0.218	0.641
Urban area	19 (51.35%)	21 (56.76%)
Rural area	18 (48.65%)	16 (43.24%)

**Table 2:** Comparison of short-term efficacy (*n* (%)).

Group	*n*	CR	PR	SD	PD	DCR (%)
SG	37	5 (13.51%)	16 (43.24%)	5 (13.51%)	11 (29.73%)	70.27% (26/37)
RG	37	2 (5.41%)	7 (18.92%)	8 (21.62%)	20 (54.05%)	45.95% (17/37)
*X* ^2^						4.497
*P*						0.034

**Table 3:** Comparison of immune function after treatment (‾*x* ± *s*).

Group	*n*	CD3^+^ (%)	CD4^+^ (%)	CD8^+^ (%)	CD4^+^/CD8^+^
SG	37	62.31 ± 5.29	42.56 ± 4.19	25.71 ± 3.62	1.57 ± 0.18
RG	37	53.62 ± 5.17	33.18 ± 3.84	31.21 ± 3.41	1.14 ± 0.14
*t*		7.146	10.039	6.727	11.470
*P*		<0.001	<0.001	<0.001	<0.001

**Table 4:** Comparison of survival period (‾*x* ± *s*, months).

Group	*n*	MPFS	OS
SG	37	4.31 ± 0.58	7.62 ± 1.34
RG	37	2.72 ± 0.41	5.48 ± 1.21
*t*		13.617	7.210
*P*		<0.001	<0.001

**Table 5:** Comparison of the incidence of adverse reactions (*n* (%)).

Group	*n*	Alimentary tract hemorrhage	Myelosuppression	Liver function damage	Skin and mucosal reaction	Thrombocytopenia	Total incidence
SG	37	2 (5.41%)	1 (2.70%)	1 (2.70%)	0 (0.00%)	2 (5.41%)	16.22% (6/37)
RG	37	3 (8.11%)	4 (10.81%)	2 (5.41%)	3 (8.11%)	4 (10.81%)	43.24% (16/37)
*t*							6.469
*P*							0.011

## Data Availability

The datasets used and/or analyzed during the present study are available from the corresponding author on reasonable request.
